# 顺应时代要求，完善我国血友病分级诊疗体系

**DOI:** 10.3760/cma.j.cn121090-20250108-00017

**Published:** 2025-04

**Authors:** 仁池 杨

**Affiliations:** 中国医学科学院血液病医院（中国医学科学院血液学研究所），国家血液系统疾病临床医学研究中心，血液与健康全国重点实验室，国家血友病病例信息管理中心，天津 300020 National Clinical Research Center for Blood Diseases, State Key Laboratory of Experimental Hematology, National Hemophilia Registry Center, Institute of Hematology & Blood Diseases Hospital, Chinese Academy of Medical Sciences & Peking Union Medical College, Tianjin 300020, China

## Abstract

作为一种罕见病，血友病是最适合进行分级诊疗的病种。中国血友病协作组在国家卫生健康委和有关部门的指导和支持下，近年来积极探索我国血友病分级诊疗体系的建设，为提高我国血友病诊疗整体水平做出了一定的贡献，也为其他疾病和学科的分级诊疗体系建设提供了可以借鉴的经验。

## Abstract (en)

As a rare disease, hemophilia is the optimal one for hierarchical diagnosis and treatment. Under the guidance and support of National Health Commission of the People's Republic of China and related organization, Hemophilia Treatment Center Collaborative Network of China has explored the establishment of the hierarchical system for the diagnosis and treatment of hemophilia in China, which contributed to the improvement of hemophilia care in China and in the meantime, shared the experience for other diseases and disciplines.

分级诊疗体系建设是国家从2012年就开始大力倡导的。国务院关于印发“十二五”期间深化医药卫生体制改革规划暨实施方案的通知（国发〔2012〕11号）明确要求“引导群众小病到基层就诊，促进分级诊疗制度形成”。国务院办公厅关于印发深化医药卫生体制改革2022年重点工作任务的通知（国办发〔2022〕14号）中再次明确要求“持续推进分级诊疗和优化就医秩序”。2024年7月18日中国共产党第二十届中央委员会第三次全体会议通过的《中共中央关于进一步全面深化改革、推进中国式现代化的决定》明确提出“促进优质医疗资源扩容下沉和区域均衡布局，加快建设分级诊疗体系，推进紧密型医联体建设，强化基层医疗卫生服务”。国家卫生健康委员会主任雷海潮于2025年1月2日在健康报发表的“贯彻落实新时代党的卫生与健康工作方针，奋力谱写健康中国建设新篇章”文章中也明确要求“以基层为重点，加快建设分级诊疗体系”。

一、血友病分级诊疗体系建设的政策保障

血友病是一种罕见病，为了改善血友病患者的医疗保障，国务院于2012年将血友病纳入大病保障和救助试点范围（国办发〔2012〕20号）。做好大病的救治和保障工作，医保是基础、医药是关键、服务是根本。为了解国内血友病患者基本信息和凝血因子类制品临床应用情况，保障医疗质量和医疗安全，为制订相关政策提供科学依据，原卫生部于2009年11月底决定建立血友病病例信息管理制度（卫办医政函〔2009〕981），指定中国医学科学院血液病医院作为国家血友病病例信息管理中心，负责全国血友病患者信息和凝血因子类制品供需信息的管理工作，协助制订相关标准和方案，并且要求各省、自治区和直辖市成立省级血友病病例信息管理中心。

为了贯彻落实国家发展与改革委员会、卫生部等六部门《关于开展城乡居民大病保险工作的指导意见》（发改社会〔2012〕2605号）和卫生部《关于加快推进农村居民重大疾病医疗保障工作的意见》（卫政法发〔2012〕74号），做好血友病医疗服务工作，原卫生部要求各地建立血友病分级诊疗体系，确定血友病定点医疗机构，成立省级血友病专家组和完善血友病病例信息管理制度（卫办医政函〔2012〕1101号）。上述政策的出台为我国血友病分级诊疗体系建设提供了保障。

二、血友病分级诊疗体系建设实践

从20世纪60年代国外就开始推广血友病患者的家庭治疗，我们现在也在国内大力推广血友病患者的家庭治疗。国内外的临床实践已经证明血友病是进行分级诊疗的最合适疾病之一。为了落实上述有关文件的要求，为推进我国血友病分级诊疗体系建设做准备，参考欧洲血友病中心标准[1]和亚太血友病防治指导原则[2]，结合我国实际情况，中国血友病协作组于2019年制订了中国血友病中心建设标准[3]。我们将血友病中心分为三个级别：综合管理中心、诊疗中心和治疗中心。综合管理中心的要求包括基本条件、科室设置与人员能力要求、基本药物配备、组织管理、检验项目及检查项目要求、诊疗及技术要求、信息化建设、继续教育及培训、学术项目、患者教育共10大项；诊疗中心的要求包括基本条件、科室设置与人员能力要求、基本药物配备、组织管理、检验项目及检查项目要求、诊疗及技术要求、信息化建设、继续教育及培训、患者教育共9大项；治疗中心的要求包括基本条件、科室设置与人员能力要求、基本药物配备、组织管理、诊疗及技术要求、信息化建设、继续教育及培训、患者教育共8大项。基于此标准我们制订了具体的血友病中心评估细则和评审标准操作规程。2020年10月在贵阳举办的第12届全国血友病大会期间，中国血友病协作组与中国罕见病联盟联合发起了中国血友病中心能力建设项目。经过为期3年的第一轮评审，截至2023年底，共有116家医院通过现场检查并获得授牌，其中综合管理中心7家、诊疗中心36家、治疗中心73家。结合在评审工作中发现的实际问题，参照2023年发表的加拿大和欧洲血友病相关组织的血友病中心标准[4]–[5]，我们对2019年的血友病中心建设标准进行更新，出台了2024年版的血友病中心建设标准[6]。截至2024年底，共有164家医院通过现场检查并获得授牌，其中综合管理中心9家、诊疗中心39家、治疗中心116家。评审工作得到了各参评医院领导和血友病多学科团队成员的高度重视与配合，也得到了当地卫生健康委员会和医保中心相关领导的支持。

除了进行现场评审，中国血友病协作组还联合中华医学会血液学分会血栓与止血学组、中华医学会骨科学分会关节外科学组等血友病诊疗涉及的多学科团队同行定期制订和更新相关指南，为国内各级血友病中心的诊疗团队提供临床诊疗的指导性文件，规范其诊疗行为[7]–[9]。这些指南的发布为实现血友病防治“属地化管理、同质化诊疗”的目标起到了很好的支撑作用。

三、思考与展望

按照我国需要接受治疗的血友病患者的患病率估算，全国约有4万余例患者需要在各级血友病中心接受治疗和得到当地医保部门的支持。每个常驻人口达到100万人的行政区域至少应该有30例左右的血友病患者需要接受治疗，这也达到了我们规定的血友病治疗中心常年随访患者例数的最低要求（至少20例）[6]。现有各级血友病中心还不能满足我国血友病患者的基本需求，我们的血友病中心建设还任重而道远。希望有志于血友病防治的各位同道继续努力，力争完成时代赋予我们的使命与责任，进一步完善我国血友病的分级诊疗体系。

中国血友病协作组在国家卫生健康委员会和有关部门的指导和支持下，近十年来积极开展分级诊疗的探索与实践，取得了一定的成绩。希望我们的实践可以为其他疾病的分级诊疗提供可资借鉴的经验。让我们大家一起为实现习近平总书记提出的“大病不出省，一般病在市县解决，日常疾病在基层解决”的目标而踔厉奋发。
